# Tuberculosis Infection in Children and Adolescents

**DOI:** 10.3390/pathogens11121512

**Published:** 2022-12-09

**Authors:** Boris Tchakounte Youngui, Boris Kevin Tchounga, Stephen M. Graham, Maryline Bonnet

**Affiliations:** 1 TransVIHMI, Institut de Recherche pour le Développement (IRD), Institut National de la Santé et de la Recherche Médicale (INSERM), University of Montpellier, 34090 Montpellier, France; 2 Department of Public Health Evaluation and Research, Elizabeth Glaser Paediatric AIDS Foundation, Yaoundé 99322, Cameroon; 3 Department of Paediatrics and Murdoch Children’s Research Institute, Royal Children’s Hospital, University of Melbourne, Melbourne 3052, Australia

**Keywords:** tuberculosis infection, children, adolescents

## Abstract

The burden of tuberculosis (TB) in children and adolescents remains very significant. Several million children and adolescents are infected with TB each year worldwide following exposure to an infectious TB case and the risk of progression from TB infection to tuberculosis disease is higher in this group compared to adults. This review describes the risk factors for TB infection in children and adolescents. Following TB exposure, the risk of TB infection is determined by a combination of index case characteristics, contact features, and environmental determinants. We also present the recently recommended approaches to diagnose and treat TB infection as well as novel tests for infection. The tests for TB infection have limitations and diagnosis still relies on an indirect immunological assessment of cellular immune response to *Mycobacterium tuberculosis* antigens using immunodiagnostic testing. It is recommended that TB exposed children and adolescents and those living with HIV receive TB preventive treatment (TPT) to reduce the risk of progression to TB disease. Several TPT regimens of similar effectiveness and safety are now available and recommended by the World Health Organisation.

## 1. Introduction

Tuberculosis (TB) is an infection caused by a bacilli of the *Mycobacterium* (*M*) *tuberculosis complex* transmitted by inhaling bacilli contained in droplets expelled by a TB contagious individual [1]. TB remains a major cause of morbidity and mortality worldwide. More than 1.5 million children and adolescents (0–19 years) fall ill with TB every year (11% of the total number of estimated cases), with the highest proportions in young children (<5 years) and older adolescents (15–19 years) [2,3]. The burden of child and adolescent TB varies between countries and regions [4]. About 78% of the estimated number of incident TB cases among children and young adolescents in 2019 and 2020 occurred in the World Health Organisation (WHO) regions of African Region (30%) and South-East Asian Region (48%); where these regions also accounted for 84% of the combined total of TB deaths in HIV-negative and HIV-positive children [2,5].

Following exposure to *M. tuberculosis*, several factors involving the host immune response will lead either to the clearance of the germ or the TB infection that can remain latent or evolve to TB disease [6]. It is estimated that 7.5 million children are infected with *Mycobacterium tuberculosis* (*M. tuberculosis*) each year and 5–10% may develop TB disease without tuberculosis preventive treatment (TPT). The risk of the development of TB disease after exposure is age-related, the cumulative risk being higher among children below 5 years of age and adolescents (15–18 years) [3,7,8,9]. The risk of progression from infection to serious disease is more frequent within the first year after exposure in children under 2 years of age, often acutely without significant prior symptoms, while in children aged between 2 and 10 years of age, it is less common and usually associated with symptoms suggestive of TB [3].

In this article, we review the risk factors of TB infection in children and adolescents as well as recent advances in diagnosis and management.

## 2. Defining TB Infection

In individuals primary infected with *M. tuberculosis*, the pathogen is completely eradicated in some, while the immune response succeeds in the containment of infection in others as some bacilli escape killing by blunting the microbicidal mechanisms of immune cells and remain in the nonreplicating (dormant or latent) state in old lesions [10]. The term “latent” defines the condition of the persistent immune response following sensitisation by *M. tuberculosis* antigens (detected through immunological tests), without clinical or radiological evidence of disease [11]. Given the challenges to define or confirm latency, the term “TB infection” (TBI) is increasingly used rather than latent TBI. This may better characterize the dynamic continuum of various states related to TB that include infection (no disease), incipient or sub-clinical disease (asymptomatic with early radiographic changes), and non-severe and severe disease states (disease clinically active). The outcome of TBI is either self-cure, latency, or disease, and the term “TB” usually refers to the disease state [6,12,13]. In high TB incidence countries, first exposure and infection commonly occur during childhood [14,15], and studies in these settings show that up to half of the adolescents have evidence of TBI [16,17]. The risk of progression to TB disease in children and young adolescents with untreated TBI is higher than in adults, with the highest age-related risk in infants and young children [2,18].

## 3. Risk Factors for TB Infection

Various and variable factors determine the risk of exposure to an index case, the risk of progression from exposure to TBI, and then the risk from TBI to disease [19]. Following exposure, the risk of TBI is determined by a combination of index case characteristics, contact features, and environmental determinants (Table 1) [3,18,19,20,21,22]. Closer exposure proximity and long hours of exposure to an infectious TB case are well-known risk factors of TBI [18,23]. The risk also increases if the index case has high bacillary disease such as sputum smear positive and/or cavitary pulmonary disease [24,25]. Household contacts of a TB index case are at high risk of TBI in all settings, irrespective of community TB incidence [24,25,26,27,28,29]. A child exposed to a person with TB in the household is almost four times more likely to have TBI than an age-matched child in the same community but without exposure to TB in the household [24]. However, a meta-analysis of the risk of TBI in children demonstrated that household contact conferred less additional risk of TBI for children aged 10–14 years compared with young children aged 0–4 years [24]. This may reflect that young children spend more time in the household compared to school-aged children and adolescents. In high TB incidence settings, the likelihood that exposure occurs outside the household increases with age and is particularly high for adolescents who have frequent social contact outside the household including in more populated or confined environments (school, public transport, place of mass gathering).

A study carried out in an especially high TB incidence township in South Africa showed an extremely high rate of TBI in childhood and adolescence, increasing with age, with a maximal rate of TBI in the mid-teenage years [30]. In an observational study carried out in a South African township, the transmission events in children outside the household occurred in transit (about 20%), school (about 20%), and other households (5–20%, depending on the child’s age). In young children (<5 years), a small proportion of transmission events (about 5%) also occurred in the workplace, possibly from parents [31]. South-African townships are places at a very high risk of TB transmission and the study findings from these settings may not be generalised to other settings. However, the information currently available on where children get infected is limited as estimates are based on data only from observational studies and mathematical modelling builds on assumptions [24,31]. Moreover, the available studies did not take into consideration several key parameters that might influence TB transmission such as the local incidence of TB (most studies conducted in very high-incidence communities) and the variability in infectiousness and age-related risks of exposure [24,31]. However, it is well-established that the household contacts of a TB index case are a high-risk group for infection and disease [32].

Among other factors, some authors have suggested that co-infection (HIV infection) and childhood illnesses (cytomegalovirus, adenovirus, and other respiratory viruses) could increase the risk of TBI and the risk of progression to disease through the impact on the host immune response to *M. tuberculosis* and/or by induced lung damage [33]. On the other hand, routine childhood vaccine could protect against TBI through an increased total IgG level [33]. Factors related to poor socio-economic status such has living in high TB incidence settings such as townships or crowded conditions also increases the risk of TB exposure and TBI in children and adolescents [20,34]. In a study among adolescents aged 12–18 years carried out in in South Africa, predictive factors for TBI were older age, ethnicity, low parental income, maternal and paternal education at the primary school level or less, current or prior household TB exposure, and absence of chronic allergic disease [16]. The characteristics of the environment can also influence the risk of exposure. Observational studies in various settings have established an association between exposure to a smoking index case and *M tuberculosis* transmission to child TB contacts [23,35,36,37,38]. Cigarette smoke induces ciliary dysfunction, reducing immune response, defects in the immune response of macrophages, and therefore may increase susceptibility to TBI [39]. Furthermore, common urban sources of indoor air pollution have been found to be associated with TBI in child TB contacts [37,40].

The Bacillus Calmette-Guérin (BCG) vaccine protects from the risk of disseminated TB and TB meningitis in young children with limited effect in old children and adults [41]. The BCG vaccine also provides some protection against the risk of TBI [42,43,44,45,46]. A recent meta-analysis found that the vaccination of newborns is effective at preventing TBI in young children, but protection does extend to adolescents [46]. A systematic review including 3855 participants estimated a pooled risk ratio for TBI in vaccinated children of 0.81 (95% CI 0.71–0.92), indicating a protective efficacy of 19% against TBI among vaccinated children after exposure compared with unvaccinated children [43].

Efforts are being made globally for the development of improved vaccines against TB. Several vaccine candidates are currently in the pipeline at different stages of clinical trials in humans [47]. Phase III trials are currently investigating the efficacy of two vaccine candidates (VPM1002 and MTBVACN3) in protecting against TB infection in infants [48,49]. Furthermore, additional TB vaccine candidates are at the preclinical stage as well as the phase 1 and phase 2 stages of development [50].

## 4. Detection of Tuberculosis Infection 

The main goal of diagnosing TBI in children and adolescents is to identify those who are at risk for developing TB who should benefit from TPT [51,52,53]. There is no test that confirms TBI and no gold standard against which to evaluate new diagnostics. The diagnosis of TBI currently relies on an indirect immunological assessment of a cellular immune response to *M. tuberculosis* antigens using immunodiagnostic testing (Table 2). There are two common tests that are used as tests for TBI diagnosis: the tuberculin skin test (TST) and *M. tuberculosis* interferon-gamma release assays (IGRAs). Although the use of both TST and IGRAs tests is recommended for the diagnosis of TBI, neither can help differentiate TBI from TB disease as they measure lasting TB immune responses that can be present in current and previous TB disease, TBI, recent or remote TB exposure, or exposure to environmental nontuberculous mycobacteria that may have cross-reactivity [54]. Therefore, the diagnosis of TBI should combine a positive immunologic TB test result and a medical evaluation to rule out TB disease (i.e., no clinical, bacteriologic, or radiographic evidence of TB disease) [55].

### 4.1. Immunologic Tests for TB Infection

The TST measures the immune reaction to an intradermal injection of tuberculin purified protein derivative (PPD) into the forearm of an individual. Several PPD formulations are currently available and used in human subjects (PPD-s, PPD S2, PPD RT23, PPD IC-65) [57], but PPD RT23 is the most widely used globally and the one recommended by the WHO and the International Union Against Tuberculosis and Lung Disease. The TST mechanism is based on a delayed-type hypersensitivity reaction cell-mediated response to tuberculin antigens usually within 48 to 72 h, corresponding to a local induration of the skin [58]. The result of the test is then read 48–72 h after the injection [57]. An induration of 10 mm or more is considered positive among children <5 years of age, or children and adolescents exposed to adults in high-risk categories, but 5 mm is the recommended threshold for immunocompromised children including severely malnourished and HIV-positive children and children with a recent TB contact [59].

TST results are affected by a complex array of factors related to a high risk for false-positive and false-negative results. False-positive TST results can be attributed to exposure to non-tuberculosis mycobacteria (NTM), BCG vaccination, error in TST administration or interpretation, and low risk of TB exposure. The positive predictive value of the TST in BCG-vaccinated children increases with age, suggesting a decrease in false-positive TST due to BCG administration over time [60]. The positive predictive value of the TST is much greater when it is applied to individuals who have a recognised risk factor for TBI [61]. The false-negative TST reaction can be attributed to recent TBI (less than 12 weeks), cutaneous anergy due to weakness of the immune system, which is common in young or malnourished children, recent live-virus vaccination or viral illness, overwhelming TB disease (military TB or TB meningitis), and error in TST administration [56,59].

The IGRA is a whole blood assay that detects the interferon gamma (IFN-γ) produced in vivo by sensitised T cells after in vitro stimulation with *M. tuberculosis*-specific antigens, namely, the 6-kDa early secreted antigenic target protein (ESAT-6) and the 10-kDa culture filtrate protein (CFP-10). IGRAs were designed to target almost exclusively *M tuberculosis* specific proteins that are not present in the BCG vaccine and the most common NTM species [62]. Nonetheless, studies have reported an association of some NTM species (*M. avium complex, M. gordonae, M. lentiflavum*) with a positive IGRA [63,64,65]. Studies carried out in low TB incidence settings suggest that IGRAs are a more specific indicator of the presence of TBI than the TST [34,66,67,68,69,70]. However, IGRA are less sensitive in younger children with a higher rate of false-negative or indeterminate results, especially in infants [71,72]. Other advantages over the TST include its interpretation, which is not user dependent and the test does not cross react with the BCG vaccine, resulting in higher specificity [34,66,67,68,69,70]. The latest IGRAs currently approved by the Food and Drug Administration (FDA) and available on the market are the QuantiFERON^®^-TB Gold in-tube (QFT-GIT™) test and the QuantiFERON^®^-TB Gold Plus in-tube (QFT-GPIT™) test, which are both based on the enzyme-linked immunosorbent assay (ELISA) technique, and the T-SPOT^®^TB test based on the ELISPOT technique, which quantifies the number of IFN-γ-producing T cells (spot-forming cells). QuantiFERON^®^-TB Gold Plus in-tube (QFT-GPIT™) is the latest generation of IGRAs and was launched with the promise of improved performance over QFT-GIT through the addition of the CD8 T-cell response [73]. However, studies directly comparing QFT-Plus with QFT-GIT in TB patients, high-risk groups, and low-risk populations have not revealed any significant improvement in its performance [74]. Further research in immunocompromised individuals and children is needed [74]. The T-SPOT^®^TB is less used, although some evidence indicates that it might produce a lower rate of indeterminate results compared to QuantiFERON-TB in children originating from the African continent and in immunocompromised children [75].

### 4.2. Considerations for TBI Testing

Dawn Nolt et al. [52] suggested an evidence based structured summarised recommendations regarding testing in children and adolescents using TST and IGRAs. Both the TST and IGRAs are imperfect methods but have high positive predictive value when applied to children with risk factors for TBI, particularly recent TB contact. Therefore, a TST or IGRA should be performed only in children with a risk factor for TBI or TB disease, in those having a disease or condition that may require significant therapeutic immunosuppression, or are suspected of having TB disease. The choice of which test to use should take into consideration the specificity and sensitivity. Studies on IGRAs show that they are more specific tests than the TST, giving fewer false-positive results, but this cannot be generalised as the majority of IGRA studies in children have been conducted in high-income countries and extrapolation to low- and middle-income settings with high background TBI rates may not be appropriate [76]. However, IGRAs have little advantage over the TST in sensitivity, and both methods are less performant in immunocompromised children. In high income settings, IGRAs are now the preferred test for immunocompetent children ≥5 years of age who have received the BCG vaccine as they provide enhanced specificity over the TST. TST has been recommended as the standard of care immunodiagnostic test in children <5 years of age by many experts [77,78,79]. Use of an IGRA in conjunction with TST has been advocated by some experts to increase the diagnostic sensitivity in this age group [80,81]. Another advantage of IGRA over TST is the shorter time interval between exposure and positivity (4 to 7 weeks vs. 2 to 12 weeks) [82,83]. Systematic reviews have suggested that IGRA performance differs in high- versus low-TB and HIV incidence settings, with relatively lower sensitivity in high TB incidence settings [84]. Additionally, IGRAs are more costly and more technically complex to perform than the TST, which is a major limitation for many high TB incidence and resource limited countries [85]. Although the basic cost of the TST is much lower compared with IGRAs, the other costs associated with the need to assess the tuberculin response at 48–72 h, both in terms of direct (transport for patients) and indirect (time for patients and health personnel) expenditures, and the conditions for the use and storage of tuberculin add to the limitation of its use [86].

The WHO recommends TBI testing whenever feasible, using either TST or IGRA independently to identify persons at highest risk for developing active TB with a preference for the TST in children less than two years of age and settings with poor laboratory infrastructure and for IGRA in persons who have received BCG vaccine and in groups that are unlikely or unable to return for TST reading [59]. This recommendation applies to all settings regardless of TB incidence threshold with implementation considerations for each setting [51]. However, in resource limited countries with an estimated TB incidence greater than 100 per 100,000 population this recommendation must take into account the operational constraints related to the availability and use of the TST and the IGRA. Therefore, considering the evidence of the benefits outweighs the harm in children and adolescents at higher risks of developing TB (children and adolescents living with HIV and child household contacts aged <5 years), the WHO recommends that in resource limited settings, TBI testing should not be a requirement for initiating TPT, particularly in settings with a high TB incidence [51].

### 4.3. Recent Developments on TB Infection Diagnosis

New innovative skin tests and IGRAs or in vitro tests for TBI testing with improved predictive values and operational characteristics are under development [87] and some are already on the market (C-Tb (Serum Institute of India, Pune, India); C-TST (formerly known as ESAT6-CFP10 test, Anhui Zhifei Longcom, Anhui, China) and Diaskintest (Generium, Moscow, Russian Federation)) [88,89,90,91]. However, published data in various populations and settings are limited for these new tests. According to a communication from the WHO [91], the new TB antigen-based skin tests (TBST) are as sensitive as TST and IGRA, making them suitable alternatives. The specificity is similar to that of IGRA and better than that of TST, particularly in populations with prior BCG vaccination history [91]. No safety signal was identified; however, regulatory evaluation for the individual products is essential before introduction of these in vivo tests. Where TST is already used, TBST implementation is expected to require some adaptation. Additionally, in many settings, TBST would be cost-saving relative to TST and IGRA. There are few data on the predictive values of these tests or on the efficacy of TBI treatment based on the results of these tests. Further research to address these gaps is needed including a comparison with TST and IGRA [91]. In September 2022, the WHO issued an updated guideline recommending the use of TBSTs for the diagnosis of TBI. Although the data were limited, the WHO supported the extrapolation of the recommendation for children and adolescents aged under 18 years including those living with HIV and those who have been vaccinated with BCG [56].

More research on biomarkers of TBI and early markers of disease development is needed. Recent promising research has reported that *M. tuberculosis* DNA can be detected in the blood of asymptomatic individuals using PCR methods with the potential to be used as a biomarker of TBI [92,93,94].

## 5. Management of Tuberculosis Infection

The aim of TBI management is to prevent the progression from TBI to TB disease and diminish the reservoir for future TB cases [50].

Randomised controlled trials have shown that TPT is effective in preventing progression to disease [95]. TB disease must be ruled out before starting TPT [96,97]. Clinical evaluation is important to identify signs and symptoms suggestive of TB and helps to exclude extrapulmonary TB [98], while chest X-rays are important to help ruling out lung parenchymal disease that could be present in asymptomatic child contacts [52]. In many settings, the use of chest X-ray is limited by the unavailability of X-ray machines, and the lack of trained health personnel to interpret radiography images [97]. The WHO therefore recommends ruling out active TB using simple symptom-based screening alone when chest radiography is not available, especially for high risk groups of child contacts <5 years of age and children living with HIV [99]. Although the sensitivity is low, symptom-based screening has a high negative predictive value to rule out active TB in child contacts, especially among those <5 years of age [100]. Computer-aided detection (CAD) technologies use artificial intelligence to detect abnormalities suggestive of intra-thoracic TB from chest X-ray [101]. Recent developments in CAD software combined with portable X-ray machine offer a potential to increase access to radiography for the screening of active TB in limited resource settings [101,102,103]. However, the CAD software would need to identify a different spectrum of radiological abnormalities than those developed for use in adults [104,105], and then performance of the CAD in child TB needs to be evaluated, especially in young children.

Considering the high risk of progression to TB disease in people living with HIV, and considering the treatment residual risk of harms, the WHO recommends TPT in all children aged ≥12 months living with HIV who are considered not to have active TB based on an appropriate clinical evaluation, regardless of TB exposure history and without prior TBI testing if not available [51]. The WHO also recommends TPT for household child and adolescent contacts with evidence of infection after ruling out TB disease by an appropriate clinical evaluation with or without chest X-ray, depending on the availability or according to national guidelines [51].

There are now several effective and safe TPT regimens (Table 3) [106]: daily isoniazid monotherapy for six months (6H), daily rifampicin plus isoniazid for 3 months (3HR), and weekly rifapentine plus isoniazid for 3 months (3HP) [51,107]. The WHO also recommends daily rifampicin monotherapy for 4 months (4R) [51]. The recently published consolidated guidelines on TPT (2020) recommend a one-month daily isoniazid-rifapentine (1HP) regimen only in individuals ≥13 years old. A recent observational cohort study has shown a good safety and feasibility of 1HP in children <13 years of age in a low-resource and HIV prevalence setting in South East Asia [108]. In settings with high TB transmission, the WHO also recommends a daily isoniazid monotherapy for thirty-six months (36H) for adolescents living with HIV. Based on the pharmacokinetic/pharmacodynamic studies in children and the safety data, the WHO recommends an increased dose of isoniazid and rifampicin in children aged <10 years (10 and 15 mg/kg/day) compared to children ≥10 years and adolescents (5 and 10 mg/kg/day). Medical providers for children and adolescents should be familiar with all of the regimens available to treat TBI to select the optimal regimen. The WHO-recommended regimens are known have similar safety and effectiveness, but the preferred characteristics include low cost, acceptability, and ease of administration to young children to result in a high completion rate [52,109,110]. Currently, 3RH is often a preferred choice for young child contacts because there is a child-friendly, dispersible and fruit-flavoured combination formulation available. The use of this formulation for 3RH under programmatic conditions in three African countries was associated with very high initiation and completion rates [111]. In contrast, the use of 3HP is still limited in young child contacts because rifapentine is still not recommended for children of less than 2 years and the current formulations require large numbers of tablets.

Despite the recent advances in the development of shorter TPT and the pragmatic WHO approach for high TB incidence and resource limited countries to reduce barriers such as chest X-ray to rule out TB disease and TBI testing before TPT initiation, there is still a low uptake of TPT among child contacts and children and adolescents living with HIV [113]. The Global TB Report 2022 shows slow progress in TPT coverage [114]. Globally, in 2021 we have reached 40% of the 5-year target in children aged under 5 years and only 3.0% of the 5-year target in older age groups for the period 2018–2022 [114]. Community-based TB household contact tracing and management approaches could be a good strategy to increase contact tracing, TB screening, and TPT management in children and adolescent household TB contacts [115,116,117]. The WHO recommends decentralised and family-centred, integrated services in children and adolescents exposed to TB [112].

## 6. Conclusions

Systematic TB screening of child contacts and children living with HIV including adolescents is essential to early detect and treat those with TB and prevent others from develop TB. This is likely to contribute to reduce the burden of TB. Any innovation that is adapted to the limited resources of most high TB incidence countries will support these efforts but the lack of tools such as chest X-ray and TBI testing should not be a barrier to prescribe TPT to these children. Innovative decentralised, child and adolescent centred approach are also essential to ensure acceptability and access to treatment.

## Figures and Tables

**Table 1:** Factors reported to be associated with TB infection in children and adolescents.

	Factors	Characteristics
1.	Factors related to the infectious case	- Infectiousness: sputum smear-positive; lung cavities on chest radiograph; frequency and severity of cough, mycobacterial strain - Intensity of exposure: duration; proximity; and conditions such as crowding and poorly ventilated dwelling
2.	Factors related to the individual	- Age (young child and adolescents) - Interaction with community - Immune condition - Bacillus Calmette-Guerin (BCG) vaccination - Presence of other medical condition (other infectious diseases) - Genotype of the host
3.	Social, environmental, and behavioural risk factors	- TB prevalence in community - Smoke, alcohol use - Indoor air pollution - Housing structure and size - Sleeping practices - Household income - Parental education - Population structure - Weather - Lower socio-economic status

**Table 2:** Comparison of currently approved T-cell-based tests including TST and IGRAs specific for *M. tuberculosis* *.

Tests	WHO Approved Technologies	How It Works	Advantages	Limitations	Last WHO Recommendations
Tuberculin skin test (TST)	PPD-S PPD S2 PPD RT23 PPD IC-65	Intradermal injection of tuberculin purified protein derivative (PPD) Type IV delayed hypersensitivity reaction within 24–72 h in individuals previous exposed to Tuberculin antigens Identified as palpable induration at the site of injection Positive result: - ≥5 mm for children HIV+, or severe malnutrition or with another severe illness. - ≥10 mm for children without these conditions (irrespective of previous BCG vaccination)	Require fewer resources compared to IGRAs More familiar to practitioners in resource-limited settings	Specificity reduced in individuals with recent BCG vaccination, those immunosuppressed and people infected with nontuberculous Mycobacteria Requires two clinic visits and is only valid if the results are read within the suggested time frame Result interpretation operator dependent Requires a cold chain Global shortages and stock-outs	TST or IGRAs equivalent options to test for TBI in all individuals TST preferred in children <2 years of age and settings with poor laboratory capacities IGRA preferred among groups unlikely to return to TST reading TBSTs recommended in children and adolescents aged under 18 years and people who have been vaccinated with BCG (conditional) WHO recommends that testing for TB infection should not be a requirement for initiating TPT among people living with HIV and child contacts aged under 5 years, particularly in countries with a high TB incidence, given that the benefits of TPT (even without testing) clearly outweigh the risk
Interferon-Gamma Release Assays (IGRAs) blood tests	QIAGEN QuantiFERON^®^-TB Gold (QFT-G™) QIAGEN QuantiFERON^®^-TB Gold in-tube (QFT-GIT™) QIAGEN QuantiFERON^®^-TB Gold Plus in-tube (QFT-GPIT™) ELISA-based WANTAI TB-IGRA Oxford Immunotec ELISPOT-based T-SPOT^®^TB (T-Spot)	Whole-blood test detecting the Interferon gamma (IFN-γ) produced in vivo by sensitised T cells after in vitro stimulation with *M. tuberculosis* specific antigens IFN-γ is released when the blood from infected individuals is incubated with the antigens; this is not the case for people without TB infection An enzyme-linked immunosorbent assay test is used to detect and quantify the amount of interferon-gamma released	Requires only a single visit and the result is available within 24 h Results are not affected by prior Bacillus Calmette–Guérin (BCG) vaccination	IGRA platforms are more expensive to run and require specialised facilities and trained personnel for testing Sensitivity limited in immunocompromised persons and in young children (QFT-GPIT™ developed to improve sensitivity in immunocompromised subjects and young children)	
New class of *M. tuberculosis* antigen-based skin tests (TBSTs)	Diaskintest (Generium, Russian Federation) Cy-Tb (Serum Institute of India, India) C-TST (formerly known as ESAT6-CFP10 test, Anhui Zhifei Longcom, China)	Use intradermal injection of antigens (ESAT-6 and CFP-10) that are specific to *M. Tuberculosis* and stimulate T-cell release of IFN-γ Type IV delayed hypersensitivity cellular immune response to *M. Tuberculosis*-specific antigens inducing a specific skin reaction in individuals previous exposed to *M. Tuberculosis*, Immune response is measured after 48–72 h as induration in millimetres	Similar accuracy to that of IGRAs (including specificity in BCG-vaccinated individuals) and greater than that of the TST Would be cost-saving relative to TST and IGRA	Limited evidence Requires a cold chain Measurement of the TBST reaction size and interpretation not standardised Global market availability limited	

HIV: human immunodeficiency virus; IGRA: interferon-gamma release assay; TB: tuberculosis; TST: tuberculin skin test; TBSTs: tuberculosis antigen-based skin tests; IFN-γ: interferon-gamma, TBI: tuberculosis infection. * Adapted from the 2022 World Health Organisation (WHO) consolidated guidelines on tuberculosis: module 3: diagnosis: tests for TB infection [56].

**Table 3:** Available treatment options for tuberculosis infection in children and adolescents *.

Treatment Duration	Regimen and Dose	Formulation and Completion Criteria	WHO Recommendations
1 month	One month of rifapentine plus isoniazid daily (1HP) ≥13 years (regardless of weight band): Isoniazid 300 mg/day Rifapentine 600 mg/day	No child-friendly formulation available No rifapentine dosing available until 13 years of age Available formulation: Isoniazid 100 mg or Isoniazid 300 mg Rifapentine 150 mg Isoniazid 300 mg + rifapentine 300 mg FDC 28 expected doses (one dose per day for 28 days) or at least 80% of the expected doses (23 doses) over a maximum period of 38 days for a complete treatment	Recommended only in >12 years old (conditional recommendation) Alternative regimen for Adolescents ≥ 13 years living with HIV on TDF, EFV, DTG, or RAL-based ART
3 months	Three months of daily rifampicin plus isoniazid (3HR) Isoniazid: <10 years: 10 mg/kg/day (range 7–15 mg) ≥10 years: 5 mg/kg/day Rifampicin: <10 years: 15 mg/kg/day (range 10–20 mg) ≥10 years: 10 mg/kg/day	Child-friendly formulation available Available formulation: Isoniazid 50 mg/rifampicin 75 mg (dispersible tablet and FCD) 84 expected doses (one dose per day for 84 days) or at least 80% of the expected doses (68 doses) over a maximum period of 120 days for a complete treatment	Recommended in all ages (strong recommendation) Preferred regimen for HIV-negative children if FDC available Alternative regimen for HIV-positive children on EFV-based ART
	Three months of rifapentine plus high dose isoniazid weekly (3HP) Weight-banded paediatric dosing from 10 kg (300 mg isoniazid and 300 mg rifapentine) up to 40 kg (using adult dose 900 mg isoniazid and 900 mg rifapentine)	No child-friendly formulation available Available formulation: Isoniazid 100 mg or Isoniazid 300 mg Rifapentine 150 mg Isoniazid 300 mg + rifapentine 300 mg FDC 12 expected doses (one dose per weeks for 12 weeks) or at least 90% of the expected doses (11 doses) over a maximum period of 120 days for a complete treatment	Recommended ≥2 years children (strong recommendation) Preferred regimen for Adolescents living with HIV on TDF, EFV, DTG, or RAL-based ART Alternative regimen for HIV-negative children aged ≥2 years (able to swallow tablets) Alternative regimen for older HIV-positive children on EFV-based ART
4 months	Four months of daily rifampicin (4R) Age <10 years: 15 mg/kg/day (range 10–20 mg) Age ≥ 10 years: 10 mg/kg/day	No child-friendly formulation available No formulation available for infants <8 kg weight 120 expected doses (one dose per day for 120 days) or at least 80% of the expected doses (96 doses) over a maximum period of 160 days for a complete treatment	Recommended as alternative regimen option in all age but there is no suitable paediatric formulation (conditional recommendation) May be an option for TPT among contacts of people with known isoniazid-resistant rifampicin-susceptible TB
6–9 months	Six or nine months of daily isoniazid monotherapy (6H) <10 years: 10 mg/kg/day (range 7–15 mg) ≥10 years: 5 mg/kg/day	Child-friendly formulation available Available formulation: Isoniazid 100 mg (dispersible tablet) 182 expected doses (one dose per day for 182 days) or at least 80% of the expected doses (146 doses) over a maximum period of 239 days for a complete treatment	Recommended in all ages (strong recommendation) Preferred regimen for HIV-positive children on LPV-RTV, NVP, or DTG Preferred regimen for HIV-negative children if FDC not available Alternative regimen for all age group
36 months	Thirty-six month of daily isoniazid monotherapy (36H)		Recommended in adolescents living with HIV in settings with high TB transmission (conditional recommendation)

1HP: 1 month of isoniazid and rifapentine daily; 3HP: 3 months of isoniazid and rifapentine weekly; 3HR: 3 months of isoniazid and rifampicin daily; 6H: 6 months of isoniazid daily; FCD: fixed-dose combination; DTG: dolutegravir; EFV: efavirenz; FDC: fixed-dose combination; RAL; raltegravir; TDF: tenofovir disoproxil fumarate; TB: tuberculosis; TPT: tuberculosis preventive treatment. * Adapted from the 2022 World Health Organisation (WHO) Operational Handbook on Tuberculosis: Module 5: Management of Tuberculosis in Children and Adolescents [112] and the 2020 World Health Organisation (WHO) Consolidated Guidelines on Tuberculosis: Tuberculosis Preventive Treatment [51].
